# Portal hypertension as a result of the incomplete surgically treated advanced alveolar echinococcosis: a case description

**DOI:** 10.1186/s12876-020-01320-0

**Published:** 2020-06-05

**Authors:** Ł. Pielok, M. Karczewski, W. Cierach, P. Zmora, E. Lenartowicz, J. Stefaniak

**Affiliations:** 1 Department and Clinic of Tropical and Parasitic Diseases, Poznan University of Medical Sciences, Przybyszewskiego Street 49, 60-355 Poznań, Poland; 2 Department and Clinic of General and Transplant Surgery, Poznan University of Medical Sciences, Przybyszewskiego Street 49, 60-355 Poznań, Poland; 3 Institute of Bioorganic Chemistry Polish Academy of Sciences, Z. Noskowskiego Street 12/14, 61-704 Poznań, Poland

**Keywords:** *Echinococcus multilocularis*, Human alveococcosis, Portal hypertension, Liver, Joidance

## Abstract

**Background:**

Infection of *Echinococcus multilocularis* causes in humans the alveolar echinococcosis. Although the infection has world-wide distribution it is rarely detected. Diagnosis of alveococcosis is difficult because of not typical clinical picture and irregular results of radiological examinations suggesting neoplasmatic process which begins in the liver tissue or in the biliary tracts. The parasitic growth is slow, so the illness is quite often established in late invasion period. Treatment of long-lasting and late diagnosed infection is difficult and requires cooperation of parasitologists together with surgeons to avoid life-threatening organ dysfunction.

**Case presentation:**

We describe a young male patient, diagnosed, according to the radiological, immunological and histological examination results, infection of *Echinococcus multilocularis*, who was treated with not radical resection of pathologic mass together with persistent albendazole intake. The right hepatectomy was performed. In addition, visible cysts were removed from the left lobe of the liver in nonanatomical resection and suspicious calcified lesions in hepatoduodenal ligament were also removed. After the operation portal hypertension, with splenomegaly and symptoms of the liver cirrhosis occurred (thrombocytopenia, collateral venous circulation, first degree varices oesophagii). The portal hypertension probably could be a result of incomplete surgery due to extended parasitic infection and liver anathomical changes due to performed procedures, because the portal hypertension and it’s further complications had not been observed before the operation.

**Conclusions:**

*Echinococcus multilocularis* should be taken under consideration in differential diagnosis of irregular lesions within the liver. Lon-lasting invasion could be responsible for the irreversible secondary liver changes such as cirrhosis and portal hypertension. The surgery treatment (treatment of choice) is difficult and it’s results depends on the invasion period the patient is operated on. After the surgery the patient requires careful follow – up, to detect early complications.

## Background

Alveolar echinococcosis, caused by the metacestode of the fox tapeworm *Echinococcus multilocularis*, is the most pathogenic zoonosis in temperate and arctic regions of the northern hemisphere [1]. Parasite transmission occurs when eggs of the tapeworm, excreted by the final hosts (usually foxes but also dogs, wolves and cats), are ingested accidentally by humans [2, 3]. In humans *E.mulitlocularis* infection is one of the reasons of liver lesions. For many years the illness is not detected because is asymptomatic. Because of increase of foxes population in Poland the risk of parasite transmission to humans is mounting [4]. Diagnosis of alveococcosis is difficult because of not typical clinical picture and irregular results of radiological examinations (ultrasound of the abdomen cavity -USG, computed tomography-CT, magnetic resonance imaging-MRI) suggesting neoplasmatic process which begins in the liver tissue or in the biliary tracts [5–7]. According to the pathologic lesions localization the PNM (primary liver location, involvement of neighbouring organs and metastatic changes) classification is used to evaluate the disease advanced [8]. Helpful in diagnostics are serology tools performed by screening ELISA- enzyme-linked immunosorbent assay method, which detects non-specific anty-Echinococcus IgG. Western-blot confirms the diagnosis, and EM2-EM18 ELISA detects very specific anty- multilocularis antibodies [9–12]. In controversial cases the diagnosis can also be confirmed after histopathology section of the liver tissue or after performing polymerase chain reaction- PCR, which detects parasite DNA fragments [13, 14]. Long asymptomatic parasite’s development causes that diagnosis is often established in advanced infection period, which delay initiation of specific treatment . All these leads to progressive organ dysfunction with full symptomatic liver cirrhosis [15].

After only several years, when the patient is not treated, cholestasis develops, thrombotic disturbances appears and changes in other distant organs [16]. These all pathologic processes as well as presence of the parasite is responsible for the full symptomatic liver fibrosis with ascites, collateral venous circulation with oesophagi varices. In such cases the patient requires combined multidrug therapy together with paliative surgical procedures (hemihepatectomy, gastroscopy, biliary tract artificial) [17] and frequent, both parasitological and surgical, follow-up. Sometimes the patient requires the liver transplantation [18, 19].

*Echinococcus multilocularis* infection should be taking under consideration in differential diagnosis in patients with non –specific liver focuses, specially suspected of neoplasmatic disorders with normal liver function tests-LFTs (GGTP, ALP, ALT, AST) [20]. Early alveococosis diagnosis and suitable treatment initiation, could protect the patient from life –threatening complications, which correlates with longer survival and better quality of life [21].

In this work we present a case of a young man with a huge pathological mass within the liver, who was diagnosed alveococcosis and treated with the not-radical operation theater together with albendazole (Zentel, GSK) intake in whom the portal hypertension occurred as a postsurgical complication.

## Case presentation

A 31-year old male patient admitted to the Tropical and Parasitic Disease Department of Poznań University of Medical Sciences, Poland, because of the presence of a tumor-like lesion within the liver. The patient had been living in a small village surrounded by forests in which a big foxes population has been detected.

Prior to the admission the patient had suffered from influenza like syndromes, pain in the right subcostal region and suddenly joidance.

He was admitted to the local Surgery Department with suspicion of biliary tract pathology. CT scan gave the evidence of irregular mass with disseminated calcifications. He was diagnosed undifferentiated hepatitis with cholestasis.

Because of atypical radiology results suspicion of *Echinococus* infection was done. ELISA serology test was positive (2.9 Units; positive above 1.0). The patient was moved to the Tropical and Parasitic Clinic in Poznań for further investigations.

On admission day the physical examination was unremarkable. Blood tests showed elevated levels of bilirubine (2 mg%), alkaline phosphatase (172-248 U/l) gamma glutamylo trans peptidase- GGTP (135-262 U/l).

**Fig. 1:**
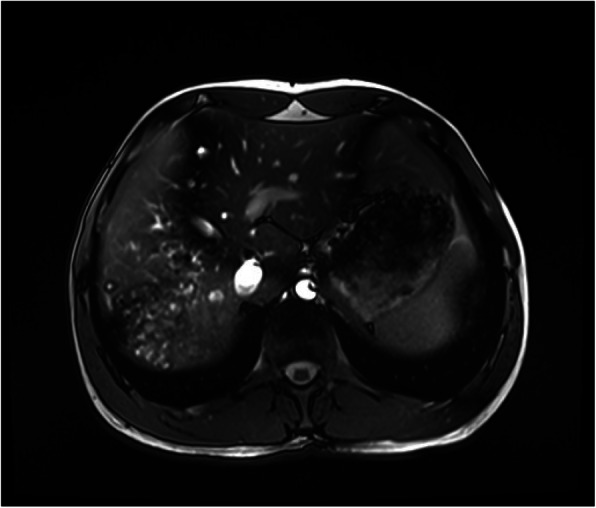
MRI of the abdomen cavity - fluid lesions and disseminated calcifications within V, VI and VII liver segments and widening of the intrahepatic biliary tracts

USG of the abdominal cavity revealed presence of a huge calcified lesion in the VII-th liver segment with the diameter of 12.3 × 2.8 cm and in the II-nd liver segment a solid hyperechogenic focus with calcifications inside as well as disseminated calcifications in the interhepatic biliary tracts neighborhood. MRI showed the liver enlargement, with irregular tissue. In the VII, VI and V segments polycyclic fluid lesion and disseminated inside the right lobe smaller fluid foci as well as biliary tract widening (Fig. 1).

According to the picturesque data suspicion of alvecococcosis was done.

ELISA test (Echinococcus IgG) was positive – 50 NTU (positive above 11NTU) and confirmed with positive Western-blot which revealed presence of specific for Echinococcus multilocularis IgG (7,16,18, 26–28 kDa). ELISA EM2-plus (anty-E.multilocularis) was also high positive (> 3.0ABS). The patient was finally diagnosed the liver alveococcosis with P2M0N0 stage.

When the diagnosis was established the albendazol therapy (2x400mg/day) was initiated together with ursodeoksycholic acid (2x250mg/day) in order to lowered bilirubine level and protect from apoptosis healthy liver tissue . The patient was qualified to the surgery treatment and was moved to the General and Transplant Surgery Department, Poznan University of Medical Sciences. MELD score of the patient was 10 (creatinine 1.13 mg/dl; bilirubin 1,12 mg/dl, INR 1,15, not dialysed).

**Fig. 2:**
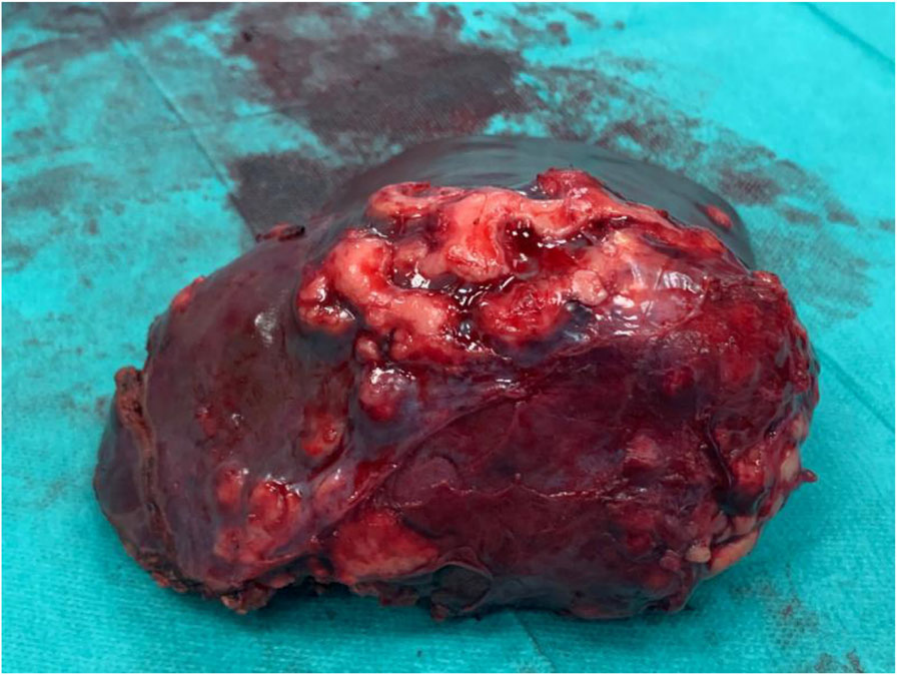
Removed enlarged liver with parasitic (*E. multilocularis*) masses

The right hepatectomy was performed. Access to the liver was achieved by bilateral subcostal incision and then mobilizing the liver from its ligamentous attachments, including the coronary ligament, and left and right triangular ligaments, then anatomic resection of the fifth, sixth, seventh and eighth liver segments was performed. Right portal vein, right hepatic artery and right hepatic duct was ligated and cutted. In addition, visible cysts were removed from the left lobe of the liver in nonanatomical resection and suspicious calcified lesions in hepatoduodenal ligament were also removed (Fig. 2). Postoperative course complicated by lymphorrhea, conservative treatment was initiated, obtaining improvement. In ultrasound on the 8th postoperative day, spleen enlargement occurs (141x55mm).

The histopathology examination of the all 3 speciments revealed presence of chronic inflammatory changes with thick wall calcified granulomas and accelular homogeny infiltrates with necrosis cavities.

Additionally, the biological material after surgery was used for molecular genotyping of *Echinococcus* sp. For this reason, the DNA was extracted from the removed liver tissue with lesions, using the commercially available kit (NucleoSpin Tissue, Macherey Nagel, Dueren, Germany). Next, the isolated DNA was used as a template in the PCR with *Echinococcus* sp. 12S rRNA-specific primers (EM-H15 and EM-H17), according to Stieger et al. (2007) [22]. Moreover, as a control of DNA quality and presence of PCR inhibitors, we used isolated genetic material to amplify human GAPDH, according to Xiang et al. (2012) [23]. Finally, the PCR product was sequenced and the obtained sequences were aligned using BioEdit software.

**Fig. 3:**
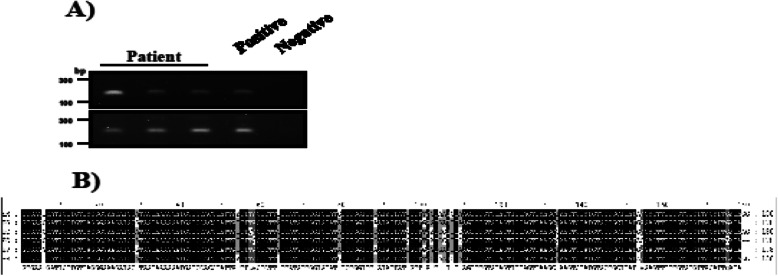
The result of PCR reaction with *Echinococcus*-specific primers, confirming that the patient was infected with *Echinococcus* sp. (Fig. 3**a**). Further sequence analysis revealed the complete similarity with *Echinococcus multilocularis* 12S rRNA (Fig. 3**b**)

Based on the PCR reactions with *Echinococcus*-specific primers, we confirmed that the patient was infected with *Echinococcus* sp. (Fig. 3a). Further sequence analysis revealed the complete similarity with *Echinococcus multilocularis* 12S rRNA (Fig. 3b), suggesting that the clinical outcome and after surgery complications were not correlated with parasite genotype and potential more pathogenic *Echinococcus multilocularis* strain.

After the operation albendazol treeatmet was continued. With no other major or minor complication patient with normal level of bilirubin, alanine transaminase and aspartate transaminase was discharge on 17th postoperative day.

**Fig. 4:**
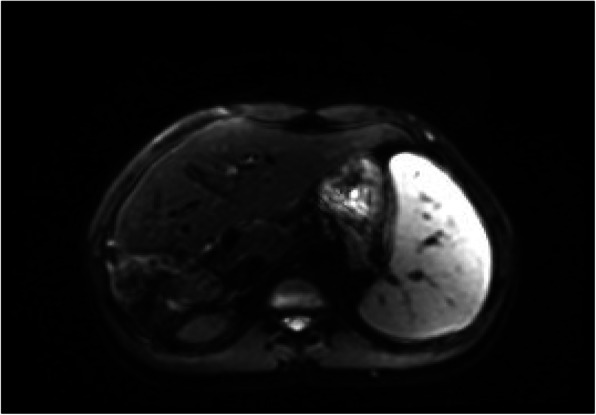
MRI of the abdomen cavity permormed after the operation – numerous calcified lesions and splenomegaly

The patient’s follow –up, performed 10 months after the operation gave the evidence of thrombocytopenia (80G/l), leucopenia (3.1G/l) and syderopenic anemia. MR of the abdomen cavity showed presence of numerous hypodensic partially calcified lesions within the remaining liver segments. Moreover critical portal vein constriction (the diameter 3-4 mm), collateral venous circulation in the liver hil were detected. The examination revealed also splenomegaly as a result of the portal hypertension (Fig. 4). Performed endoscopy of upper gastrointestinal tract revealed presence of first degree varices oesophagi as well as gastritis and duodenitis.

Recently, patient had control MRI (06.03.2020) and there are no active outbreaks of alveococosis in the liver parenchyma, compared to previous studies, the image is stable. Collateral vessels are visible in the liver cavity. The spleen is enlarged, by a maximum length of about 16 cm (before operation spleen length was 14 cm). Patient is clinically asymptomatic. But he still requires regular, every 6 months follow-up in the Tropical and Parasitic Clinic.

## Discussion and Conclussions

Infection caused by *Echinococcus multilocularis* is the most dangerous parasitic zoonosis in Europe. Left untreated, it has a very high mortality rate after located in vital organs such as liver, lungs, brain [24]. The alveolar cyst causes a maling tumor-like lesions with infiltrative, proliferative and destructive character, which locates in the liver primarily, then metastasizes to the other organs [25]. *Echinococcus multilocularis* is a parasite with capabilities of mass-forming affect neural and vascular invasion. Within the liver, the parasite’s unrestricted growth is responsible for focal calicifications and finally the whole necrosis. Due to vascular and neural invasion Buddi-Chiari syndrome may be the clinical presentation of this condition [26].

Disseminated forms of alveococcosis appears to be associated with invasive growth of the parasite and the related necessity to perform vast resection of the liver and adjacent organs, as well as resection and prosthetic repair of the major vessels [17].

Long lasting and late diagnosed alveococcosis leads gradually to the liver dysfunction with its internal structure rebuilding. Consequences of this are changes in the portal vein circulation and liver cirrhosis [16]. Hepatofibrosis as a result of the parasite-host interaction is a pathological feature of *Echinococcus multilocularis* infection that destroys normal liver tissue leading to the portal hypertension and the formation of peripheral fiberboards around the metacestode is a major reason of clinical symptoms appearance [27, 28].

To avoid serious and often irreversible life-threatening for the patient complications the diagnosis of *Echinococcus multilocularis* infection should be early obtained and surgical treatment should be immediately initiated, because delayed surgery gives inadequate results. Surgical treatment can be radical or sparing. Radical procedures are associated with a lower risk of relapse but they carry a greater risk of postsurgical complications. Moreover, in advanced alveococcosis only palliative operation can be performed to improve quality of the patient’s life. Postsurgical frequency of recurrences is connected to incomplete parasite’s excision [29]. Lately,in literature you can find descriptions of new surgical techniques such as associating liver partition and portal vein ligation, which can be safely in young individuals [30].

Described, minimally invasive surgery, the laparoscopic and robotic approach which require high expertise in liver surgery [31] could be suitable in treatment of small focci of *Echinococcus multilocularis*. But in advanced cases with parasites dissemination laparatomy used to be the technique of choice.

In the described above case the portal hypertension probably could be a result of incomplete surgery due to extended parasitic infection and liver anathomical changes due to performed procedures, because the portal hypertension and it’s further complications (thrombocytopenia, splenomegaly) had not been observed before the operation. Moreover, the results of the molecular investigastions suggest that the clinical outcome and after surgery complications were not correlated with parasite genotype and potential more pathogenic *Echinococcus multilocularis* strain. Data showed in this manuscript proofed that *Echinococcus multilocularis* should be taken under consideration in differential diagnosis of irregular lesions within the liver. Long-lasting invasion could be responsible for the irreversible secondary liver changes such as cirrhosis and portal hypertension. The surgery treatment (treatment of choice) is difficult and it’s results depends on the invasion period the patient is operated on. After the surgery the patient requires long life albendazol intake and careful follow – up, to detect early or late complications.

## Data Availability

Not applicable.
